# A new radiographic view of the hindfoot

**DOI:** 10.1186/1757-1146-6-48

**Published:** 2013-12-13

**Authors:** Kazuya Ikoma, Masahiko Noguchi, Koji Nagasawa, Masahiro Maki, Masamitsu Kido, Yusuke Hara, Toshikazu Kubo

**Affiliations:** 1 Department of Orthopaedics, Kyoto Prefectural University of Medicine, Kawaramachi-Hirokoji, Kajii-cho, 465, Kyoto 602-8566, Japan; 2 Department of Orthopaedic Surgery, Tokyo Women’s Medical University, Tokyo, Japan

**Keywords:** Hindfoot, Alignment, X-ray

## Abstract

**Background:**

A new radiographic view was proposed to evaluate the coronal plane alignment of the hindfoot under weightbearing conditions.

**Methods:**

We studied 46 feet of individuals with normal appearing asymptomatic feet. On the antero-posterior roentgenogram using this new method, the line from the top of the sustentaculum tali to the lateral-inferior end of the posterior articular surface of the talus was obtained as the standard line showing varus or valgus of the calcaneus. We defined the angle between the longitudinal axis of the tibia and the standard line as described above as Varus-Valgus angle (V-V angle).

**Results:**

The mean (±SD) V-V angle of the 46 feet studied was 76.4 (±3.6) degrees.

**Conclusion:**

The findings from this study indicate that it is possible to estimate the alignment of the hindfoot quantitatively by comparing individuals to the mean V-V angle that we calculated in our sample, which was 76.4 degrees.

## Background

Accurate evaluation of the alignment of the hindfoot in the coronal plane is essential in the assessment and treatment of hindfoot pathological conditions. Cobey’s method utilising x-ray measurement and its modified procedure are often used to estimate the hindfoot coronal alignment [1-5]. A previous study found a good correlation between clinical evaluation and measurements of hindfoot alignment [6]. However, these x-ray methods do not clearly show the hindfoot, so coronal alignment is difficult to evaluate precisely [7,8]. Therefore, the aim of this study was to describe and evaluate a new method of radiographically imaging the coronal plane alignment of the hindfoot that could quantitatively estimate this alignment under weightbearing conditions.

## Methods

### Participants

This study was approved by Institutional Review Board Kyoto Prefectural University of Medicine. A total of 40 participants (twenty men and twenty women) were recruited. All gave informed consent to participate in the study. All participants had no history of discomfort in the foot. Plain film weightbearing radiographs (dorso-plantar view and lateral view) were taken for all volunteers to initially exclude cases of pes planus and pes cavus that could have coronal malalignment. Twenty-three participants with normal hindfoot alignment subsequently went on to participate in this study, including 20 men and 3 women. The age of participants ranged from 21 to 55 years, with an average of 27.7 years. Both feet were studied, so 46 feet of the 23 participants with asymptomatic feet and in which alignment appeared normal were included.

### Radiographic technique

**Figure 1:**
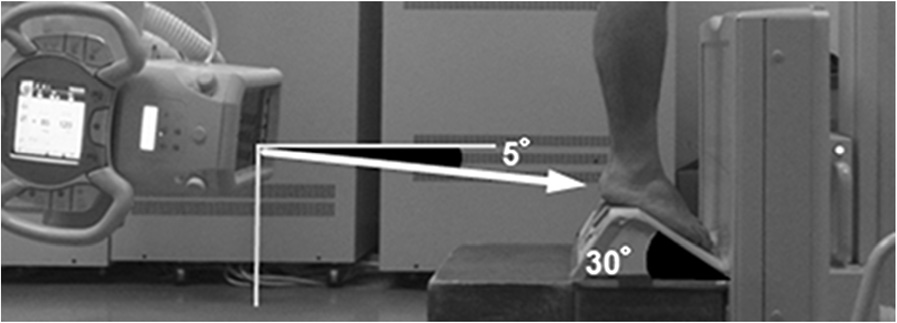
**Our radiographic technique.** Participants stood on a radiolucent platform with equal weight on both feet. This platform was flat in the rear part and inclined by 30 degree in the front part, so that the midfoot and forefoot of participants was planter-flexed. The x-ray beam was oriented down 5 degree from the horizontal.

Participants stood on a specially designed foot stand (Figure 1) with equal weight on both feet. The hindfoot was flat and the forefoot planterflexed 30 degrees on the foot stand. An x-ray film was oriented perpendicular to the floor at the front of the feet. The x-ray tube was oriented 5 degrees from the horizontal. The x-ray beam was directed from posterior to anterior 5 degrees toward the caudal side from a distance of 120 cm. The beam was centered at the level of the ankle, and the field of exposure included from the distal one third of the tibia to below the calcaneus. The ankle and the posterior subtalar joint can be clearly projected by the inclination of the x-ray beam at 5 degrees. Exposure was 80 KV and 500 mA for an exposure time of 20 msec. An x-ray filter was positioned on the source of the x-rays to regulate the exposure (i.e. darkness) from the tibia to the metatarsal bones and to image the hindfoot clearly.

### Radiographic measurements

**Figure 2:**
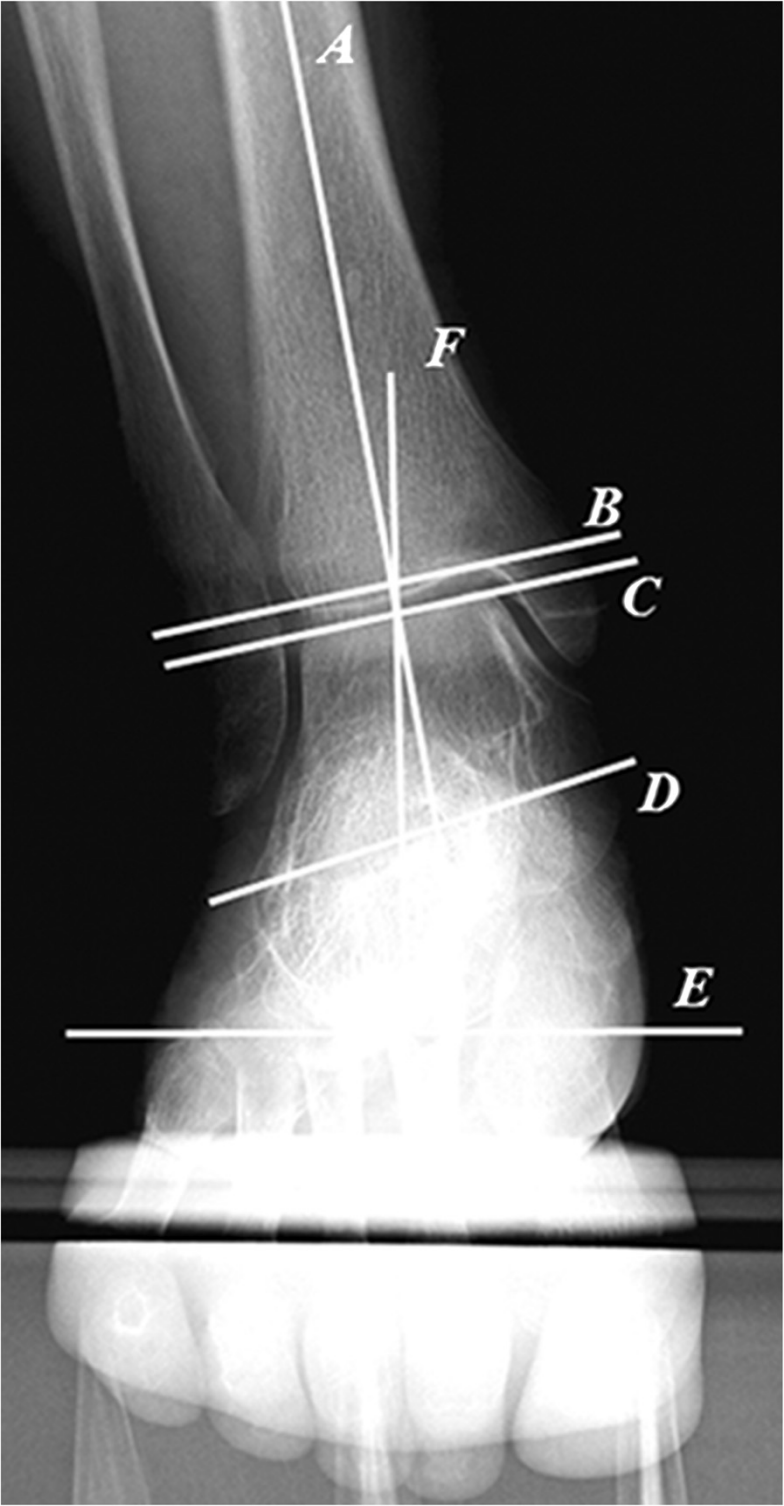
**Hindfoot alignment view.** The ankle joint and the middle and posterior subtalar facets are visualised clearly. ***A*** shows the axis of the tibia. ***B*** shows the surface of the distal tibia, and ***C*** shows the surface of the proximal talus. ***D*** shows the line from the top of the sustentaculum tali to the lateral-inferior end of the posterior facet of the calcaneus. ***E*** shows the horizontal line through the contact point of the heel. ***F*** shows the line from the cross point of ***A*** and ***C*** to the contact point of the heel.

The method of the x-ray measurements is shown on the Figure 2. *A* shows the axis of the tibia, which lays between midpoints of the tibial shaft, at approximately 8 cm and 13 cm above the ankle joint for an average foot. *B* shows the joint surface of the distal tibia and *C* shows the surface of the proximal talus. *D* shows the line from the top of the sustentaculum tali to the lateral-inferior end of the posterior facet of the calcaneus. *E* shows the horizontal line through the contact point of the heel. *F* shows the line from the cross-point of *A* and *C* to the contact point of the heel (i.e. on line E). We measured: the angle between *A* and *D* (V-V angle, the standard angle showing varus or valgus of the calcaneus relative to the tibia); the angle between *A* and *B* (A-P mortise angle: A-P mortise angle of the ankle relative to the tibia); and the angle between *A* and *F* (T-H angle: the angle between the tibia and the hindfoot).

## Results

**Figure 3:**
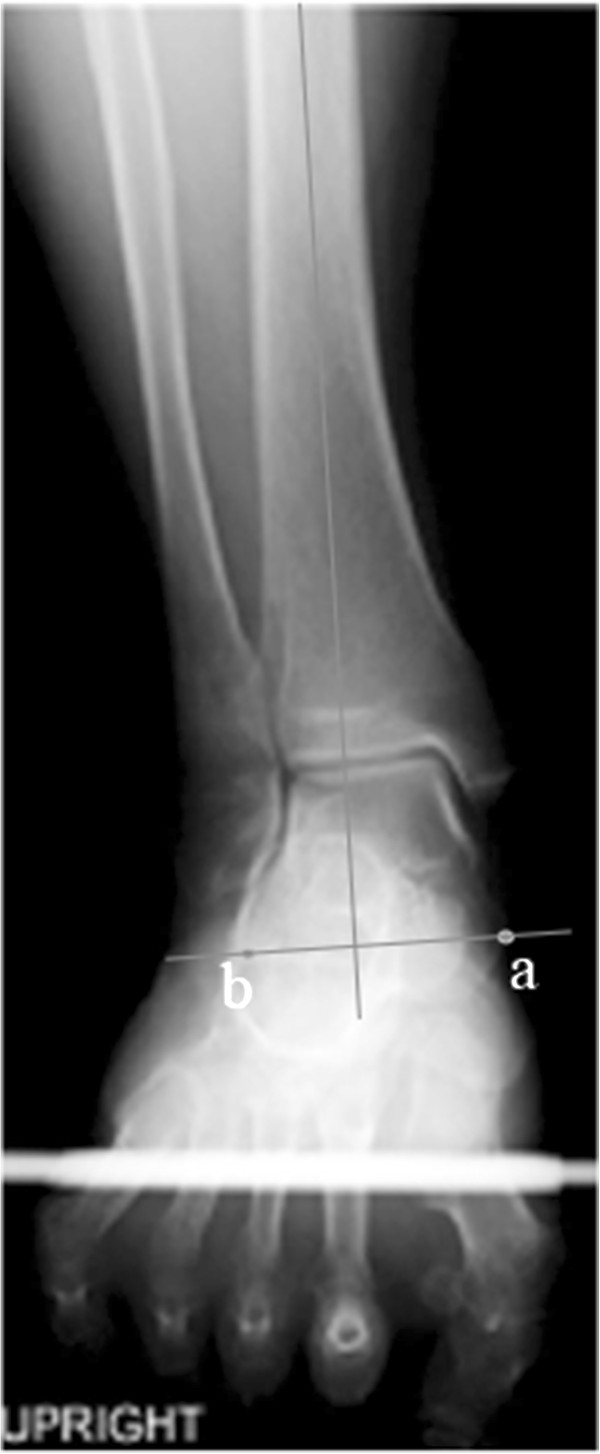
**Varus-Valgus angle: *a *** **shows the top of the sustentaculum tali and *b *** **shows the lateral-inferior end of the posterior facet of the calcaneus.**

The ankle joint, sustentaculum tali, and the lateral-inferior end of the posterior facet of the calcaneus were clearly shown on the single x-ray view taken using this new method. As indicated previously, the line from the top of the sustentaculum tali to the lateral-inferior aspect of the posterior facet of the calcaneus was designated as the standard line showing varus or valgus of the calcaneus (Figure 3). The angle between this line and the axis of the tibia was defined as Varus-Valgus angle (V-V angle). The mean (±SD) V-V angle of the asymptomatic 46 feet was 76.4 (±3.6) degrees. The average A-P mortise angle was 88.8 (±1.8) degrees and the average T-H angle was 1.5 (±2.1) degrees.

### Illustrative case presentations

The following cases are not participants in the study but highlight the typical hindfoot deformities, pes planovalgus and pes equinovarus. Furthermore, Case 1 shows correction of the coronal alignment of hindfoot after surgery.

#### *Case 1 (Figures* 4 *and *5*)*

**Figure 4:**
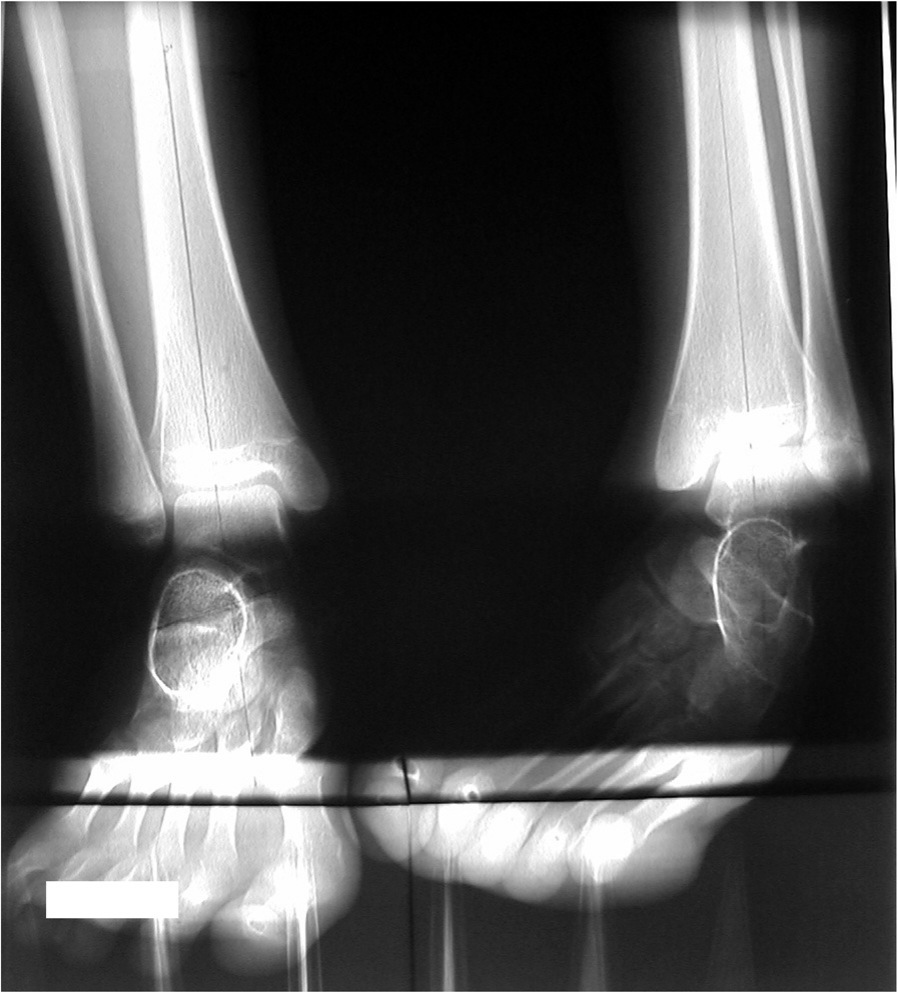
**A 14-year-old female with pes equinovarus: preoperative radiograph.** The V-V angle of the right foot was 79 degrees, while the V-V angle of the left foot was 44 degrees.

**Figure 5:**
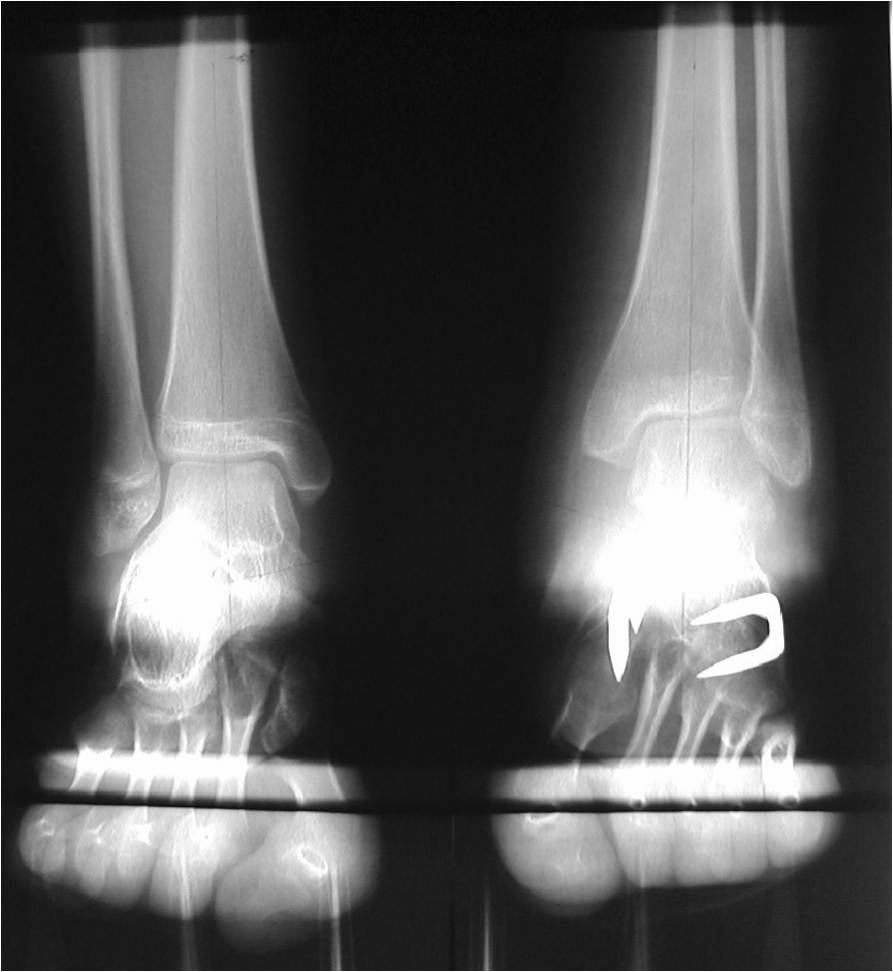
**A 14-year-old female with pes equinovarus: postoperative radiograph.** The V-V angle of the left foot corrected to 75 degrees.

This case is of a left pes equinovarus deformity in a 14-year-old female. The V-V angle of the right foot was 79 degrees, while the V-V angle of the left foot was 44 degrees (Figure 4). The V-V angle of the left foot was less than that of the right, indicative of pes equinovarus. The patient received an operation that utilised a V-osteotomy (Japas osteotomy) of the midfoot and an Achilles tendon lengthening was performed for the pes equinovarus and pes cavus deformities. Following the operation, the V-V angle of the left foot increased to 75 degrees, which is close to the average found in this study (76.4 degrees) (Figure 5).

#### *Case 2 (Figures* 6 *and *7*)*

**Figure 6:**
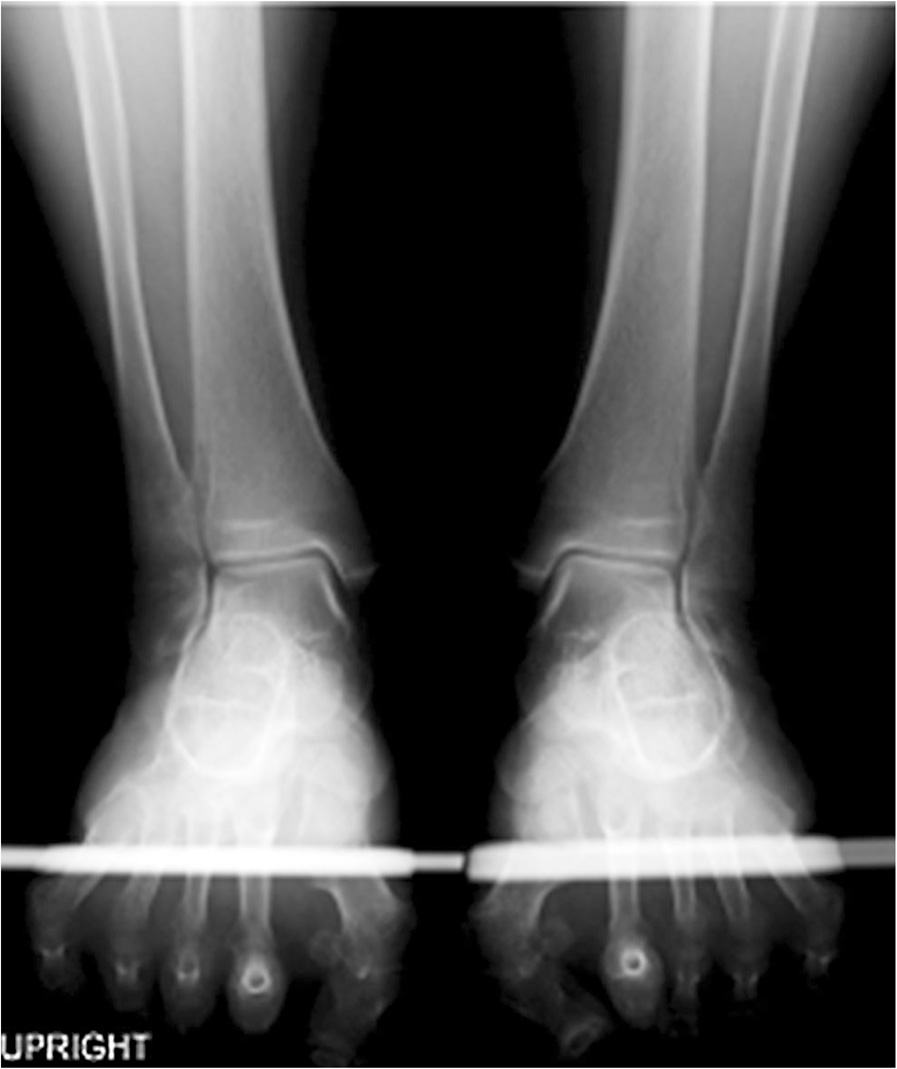
**A-66-year-old female with pes planovalgus: our hindfoot x-ray image.** The V-V angle of the right foot was 83.4 degrees, while the V-V angle of the left foot was 81.3 degrees.

**Figure 7:**
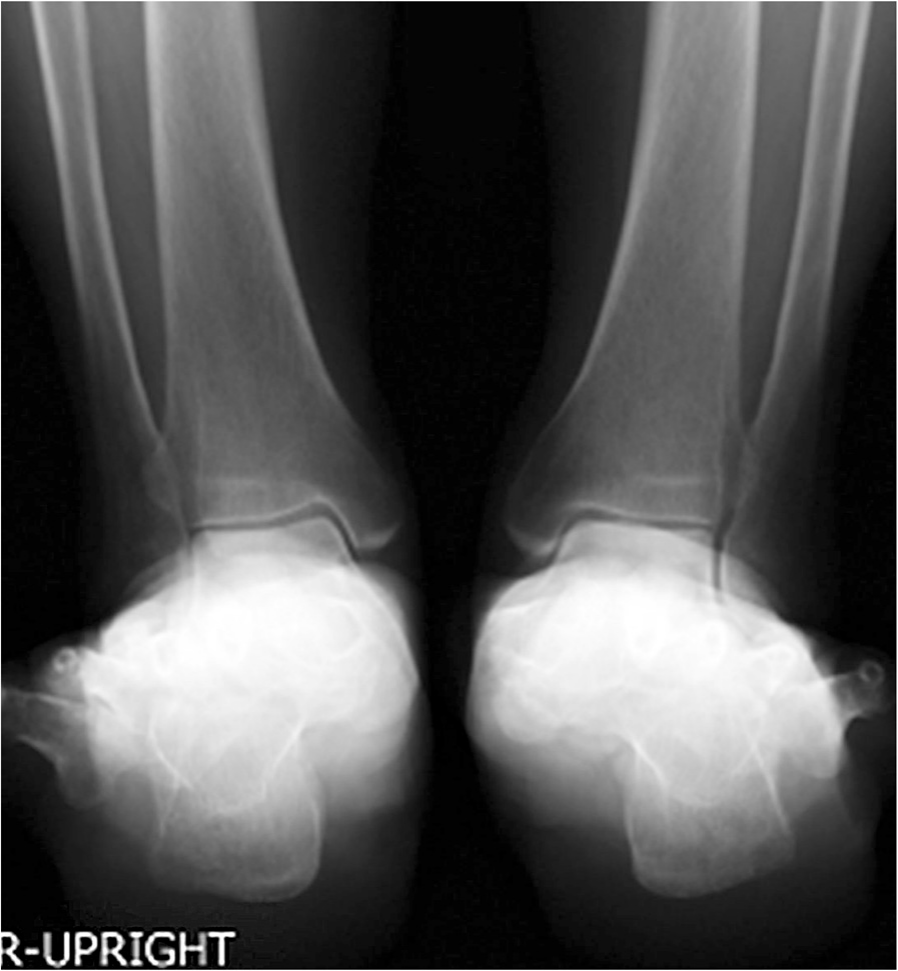
**A-66-year-old female with pes planovalgus: Cobey’s view.** The posterior facet of talocalcaneal joint remained hidden from view on the Cobey’s view.

This case is of bilateral hallux valgus deformity and pes planovalgus deformity in a 66-years-old female. The V-V angle of the right foot was 83.4 degrees, while the V-V angle of the left foot was 81.3 degrees (Figure 6). Interestingly, on a Cobey’s view, the posterior facet remained hidden from view due to superimposition of the metatarsal bones and phalanges (Figure 7), which highlighted the advantage of our technique.

## Discussion

Cobey’s view or an A-P view of the ankle under weightbearing conditions has been used in the past for evaluating the alignment of the hindfoot. However, our new x-ray view shows the hindfoot more clearly than either of those methods. Using Cobey’s method, Saltzman and colleagues evaluated the alignment of the hindfoot by measuring the horizontal distance between the axis of the tibia and the contact point of calcaneus [5]. However, it is hard to assess the tilt of the calcaneus to the axis of tibia, because the calcaneus curves, making it difficult to determine a longitudinal axis. Tanaka and co-workers evaluated the alignment of the subtalar joint using their original x-ray technique [9], finding that the ankle joint and the middle and posterior facets of the subtalar joint were depicted with this technique. Although our new x-ray method cannot evaluate the alignment of the subtalar joint, it can clearly image the ankle and the hindfoot and quantitatively evaluate the alignment of the calcaneus. Interestingly, the A-P mortise angle in our study was 88.8 (±1.8) degrees, which is close to the measured value of the A-P view of the ankle joint under weightbearing conditions reported previously as 87.7 (±3.0) degrees [10], which indicates that the position we chose to position the foot is similar to that of a standard weightbearing AP view of the ankle.

We defined the angle between the axis of the tibia and the standard line, as described above, as the V-V angle. We believe that we can evaluate the alignment of the hindfoot quantitatively by comparing the V-V angle with the mean value of 76.4 degrees that we found in our sample in this study. Our new x-ray method could be clinically applied to the quantitative evaluation of the valgus deformity of the hindfoot in the pes planovalgus deformity, and as an evaluation of the angle of the hindfoot deformity as corrected operatively (similar to Case 1). However, further research is needed to determine if this new x-ray method is useful for infants and children.

The findings of this study need to be considered in light of several limitations. Firstly, there were many more men in our sample than women, however in our volunteers many female volunteers did not meet the inclusion criteria of having a normally aligned rearfoot. Therefore, future research should measure the angle in a sample that contains more women than we managed to recruit. Secondly, we thought that there may be a problem with position of the forefoot in the x-ray technique we described due to plantar flexion of the forefoot on the rearfoot. This arose in all cases, but we did not consider this to be a problem in visualising all the required anatomical landmarks. However, future research is necessary to investigate whether there is a similar tendency with valgus and varus cases of the hindfoot compared to feet with normal hindfoot alignment.

## Conclusions

Cobey’s method or its modified procedure has often been used to evaluate the coronal plane alignment of the hindfoot. Although these views can show the angle between the weightbearing axis and the most inferior aspect of the calcaneus, the degree of tilting of the calcaneus itself to the weightbearing axis is not clear. However, the new method that we have described and evaluated in this study for radiographically imaging the hindfoot coronal alignment can more clearly depict details of the hindfoot. Our findings suggest that hindfoot alignment in patients when measured using our technique can be compared with the V-V angle described in this study. In our asymptomatic sample of participants with normal hindfoot alignment, we found a mean V-V angle of 76.4 degrees.

## Competing interests

For this manuscript, “A new radiographic view of the hindfoot” all authors state that they have no financial or personal conflicts of interest that could influence the submitted work.

## Authors’ contributions

MN and KI organised this study and drafted the manuscript. KN, KI, KM, and YH participated in the design of the study, measured the alignment, and performed the statistical analysis. All authors read and approved the final manuscript.
